# Pancreas and gallbladder agenesis in a newborn with semilobar holoprosencephaly, a case report

**DOI:** 10.1186/s12881-017-0419-2

**Published:** 2017-05-19

**Authors:** Robert Hilbrands, Kathelijn Keymolen, Alex Michotte, Miriam Marichal, Filip Cools, Anieta Goossens, Peter In’t Veld, Jean De Schepper, Andrew Hattersley, Harry Heimberg

**Affiliations:** 1 Diabetes Research Center, Vrije Universiteit Brussel, Laarbeeklaan 103, Jette, 1090 Brussels Belgium; 2 Center for Medical Genetics, Reproduction and Genetics, Reproduction Genetics and Regenerative Medicine, Universitair Ziekenhuis Brussel, Jette, Belgium; 3 Department of Pathology, Universitair Ziekenhuis Brussel, Jette, Belgium; 4 Department of Pediatrics, Universitair Ziekenhuis Brussel, Vrije Universiteit Brussel, Jette, Belgium; 5 Institute of Biomedical and Clinical Science, University of Exeter Medical School, Exeter, UK; 6 Diabetes Clinic, Universitair Ziekenhuis Brussel, Brussels, Belgium

**Keywords:** Premanent neonatal diabetes mellitus, Pancreas agenesis, Holoprosencephaly

## Abstract

**Background:**

Pancreatic agenesis is an extremely rare cause of neonatal diabetes mellitus and has enabled the discovery of several key transcription factors essential for normal pancreas and beta cell development.

**Case presentation:**

We report a case of a Caucasian female with complete pancreatic agenesis occurring together with semilobar holoprosencephaly (HPE), a more common brain developmental disorder. Clinical findings were later confirmed by autopsy, which also identified agenesis of the gallbladder. Although the sequences of a selected set of genes related to pancreas agenesis or HPE were wild-type, the patient’s phenotype suggests a genetic defect that emerges early in embryonic development of brain, gallbladder and pancreas.

**Conclusions:**

Developmental defects of the pancreas and brain can occur together. Identifying the genetic defect may identify a novel key regulator in beta cell development.

## Background

Neonatal diabetes mellitus is defined as a rare form of diabetes diagnosed before 6 months of age. It is monogenic in origin and mutations in the *KCNJ11* gene are the most frequent cause, resulting in normal pancreas and beta cell development but impaired insulin secretion [1]. Patients with mutations in the *KCNJ11* and *ABCC8* genes - encoding for the subunits of the beta-cell channel - can be treated successfully with high dose sulfonylurea instead of insulin treatment [2]. Over the past decade, more genes have been identified as a direct cause of neonatal diabetes [3–5]. Many of these genes are crucial for beta cell function and some may play a role in embryonic development of the exocrine and/or endocrine pancreas. The more upstream affected genes are in the developmental pathway, the more severe the malformation, explaining the various levels of developmental defects described in the literature [5].

Complete agenesis of the entire pancreas has only been described in very few cases. Pancreas agenesis is genetically heterogeneous. The first genetic aetiology described was caused by recessive mutations in the pancreatic duodenal homeobox gene 1 (PDX1/IPF1) [6], a transcription factor necessary for development of pancreas precursors. Mutations have also been identified in pancreas-specific transcription factor 1A gene (PTF1A) [7] and in a specific downstream enhancer [8], GATA-binding protein 6 gene (GATA6) [9] and GATA4 [10]. These genes are all acting downstream of PDX1/IPF1 [7, 10–12]. Importantly, in many cases, targeted disruption of these genes in mice replicates the phenotype of disturbed pancreatic development as seen in humans [6, 13–15]. Disorders of human pancreas development may also affect embryogenesis of the gallbladder [9, 16]. However, the frequency of gallbladder agenesis, its clinical implications and its causes remain unknown.

Holoprosencephaly (HPE) is the most common structural anomaly of the developing forebrain characterized by incomplete separation of the prosencephalon and occurs in about 1 in 8.000 living births [17]. The defects associated with HPE occur very early in embryonic development, at the stage of gastrulation. The etiology is heterogeneous and remains incompletely understood. A number of environmental factors have been implicated as well as genetic defects including both chromosomal aberrations and mutations in four genes (*SHH*, *SIX3, ZIC2* and *TGIF*). However, mutations in these genes can only be identified in 25% of patients with normal chromosomes suggesting that additional susceptibility genes remain to be identified [17].

Patients with concurrent developmental disorders in brain and pancreas, including neonatal diabetes, have been reported [7, 18, 19] suggesting a link between embryogenesis of both organs. One report of particular interest describes a family with three subjects affected by cerebellar agenesis and neonatal diabetes [20] who had homozygous mutations in *PTF1A*. Additional studies in Ptf1a null-mutant mice confirmed its causal relationship with the phenotype (6). These reports convincingly demonstrated the critical role of transcription factors common to both neuronal and pancreatic development.

In the current report we describe for the first time a patient with both HPE and neonatal diabetes mellitus. HPE, pancreatic and additional gallbladder agenesis were confirmed by autopsy. Considering the reported mechanistic evidence of neonatal diabetes and the striking similarity with the reported PTF1a mutation -which is not the causative factor in this case- a monogenic origin is suspected.

## Case presentation

A few hours after birth a Caucasian female was transferred to the neonatal care unit of UZ Brussels for bradycardia and recurrent hypoxia. The girl was born after a pregnancy of 38 weeks and was small at gestation (1100 gram). Pregnancy was uneventful besides intra-uterine growth retardation that could not be attributed to any specific cause. The mother had been healthy throughout the entire pregnancy and did not use medication, drugs or alcohol. Delivery was by uncomplicated caesarean section and Apgar score was 9/10/10. Seven years earlier, the mother successfully gave birth to a girl with intra-uterine and postnatal growth retardation (1.88 kg body weight at birth, 14 kg at age 8) but was otherwise normal. Both the mother and father are healthy individuals of relatively short stature (148 and 156 cm respectively) without a family history of diabetes mellitus, mental retardation or structural brain anomalies such as holoprosencephaly. The parents are not consanguineous.

Physically, the newborn appeared dystrophic with receding forehead, cylindrical nose, mild hypotelorism, dysplastic left ear, hypoplastic zygomatic bone and thumbs in adduction. Structural limb anomalies including polydactyly were absent, palate and lips normal. There were clear distinct female genitalia and the anus and rectum were normal.

Neonatal diabetes was diagnosed within 24 h after birth with blood glucose values exceeding 500 mg/dL and undetectable C-peptide values (<0.03 nmol/L). The diagnosis was not associated with ketoacidosis. Treatment consisted of intravenous insulin (1-2 IU/24 h) for 20 days after which the patient was switched to subcutaneous insulin NPH twice daily (0.5 IU twice daily). Despite intensive monitoring, glycaemic control was difficult with both hyper- and hypoglycaemia. On admission the patient received parenteral nutrition. Enteral nutrition was introduced at day 7. On this additional exocrine pancreatic insufficiency was suspected by flat, yellowish stool in which trypsin could not be detected and low amylase and lipase activity in the blood. From day 14 onwards the infant only received enteral feeding together with oral pancreatic enzymes (Creon®) that was well tolerated and resulted in weight gain. Complete absence of the pancreas was confirmed by magnetic resonance imaging. The gallbladder was not visualized but there was no evidence of jaundice. Brain ultrasound revealed the absence of the corpus callosum and immature brain development. Magnetic resonance imaging confirmed semilobar holoprosencephaly. Tests for inborn errors of metabolism showed no abnormalities and no clinical syndrome could be identified. At this point, besides a prominent sternum no additional malformations where found.

### Analysis of patient DNA

Genomic DNA was isolated from peripheral blood samples of the patient to perform G-banded karyotype, and FISH analysis of the subtelomeric regions, as well as for further molecular genetic sequencing. IPF1, PTF1A, GATA6, GATA4, HNF1B and HNF6 were sequenced using a tiled targeted capture method followed by next generation direct sequencing using previously described methodology [21, 22]. This detects both large deletions and duplications.

G-banded karyotype was performed on peripheral leucocytes as well as on cultured skin fibroblasts and was normal. The subtelomeric chromosomal regions were investigated by fluorescent in situ hybridisation (FISH with TelVision probes, Vysis©) to exclude submicroscopic deletions or duplications, and this was equally normal. No mutations could be identified in all the genes known to be associated with pancreatic aplasia or neonatal diabetes including *IPF1, PTF1A, GATA6, GATA4, HNF1B*, and *HNF6*. In spite of intensive insulin and pancreatic enzyme replacement, the patient died 12 weeks and 3 days after birth.

### Autopsy

Informed consent from the parents was obtained before autopsy. Experienced pathologists performed the autopsy within two hours after death. After examination of the external body the internal abdominal and thoracic organs were procured and examined. To allow removal of the brain, a neuropathologist opened the skull. The pituitary gland was carefully removed from the sella turcica and the brain conserved in 10% formol. Coronal brain sections were performed two months following conservation to examine macroscopic morphology.

**Fig. 1:**
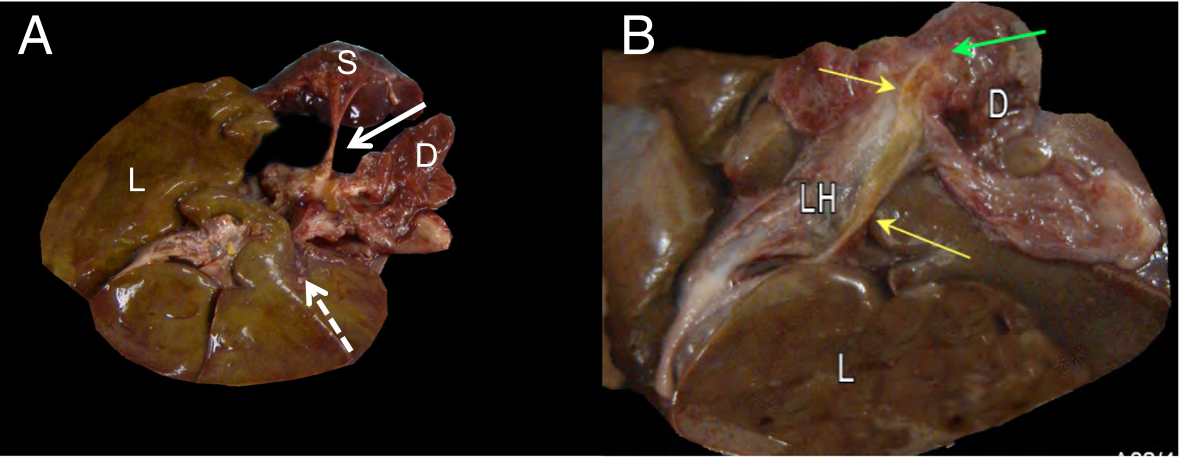
**a** No pancreatic tissue could be identified on the orthotopic location along the splenic vessels (*white arrow*) nor gall bladder (*dashed arrow*). **b** View at het choledocus (*yellow arrows*). No ductus cysticus nor gall bladder. The choledocus ends in the duodenum (D) via Vater’s Ampulla (*green arrow*). Wirsung duct an ventral pancreas are absent. L: Liver, LH: liver hilum, S:spleen

**Fig. 2:**
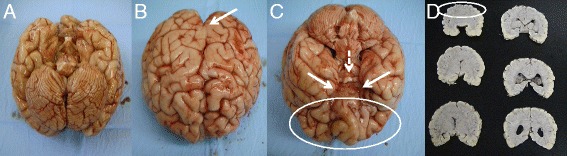
**a** Development of the cerebellum appears normal. **b** Both hemispheres are fused up to the gyrus centralis (*arrow*) with presence of polymicrogyria. **c** Fusion of the anterior brain is encircled. Bulbus olfactorius is bilaterally absent (*arrows*) while the chiasma opticum is normal (*dashed arrow*). **d** Sequential coronal sections of the brain demonstrate the incomplete separation common to semilobar HPE. Inter-hemispheric fissure is invisible in the anterior brain (region encircled). The ventral horns of the lateral ventricle are absent, as well as the genu of the corpus callosum

At autopsy the liver, spleen, stomach and duodenum were removed and examined separately. No orthotopic pancreas tissue was observed (Fig. 1a). After localisation of Vater’s ampulla the common bile duct was found but pancreatic duct, cystic duct and gallbladder were absent (Fig. 1b). A small amount of accumulation of biliary pigment in the liver was found but intra-hepatic biliary ducts and liver architecture appeared normal (Fig. 1). Heart, lungs, kidneys, adrenal glands, ureters, bladder, ovaries, uterus and muscle were normal both macro- and microscopically. The duodenum was permeable without atresia, obstruction or stenosis of the small intestine. Macroscopic analysis of the brain showed a normal cerebellum (Fig. 2a) but polymicrogyria and partial fusion of the frontal lobes were obvious (Fig. 2b and c). Temporal and occipital lobes and brainstem were normal. Rhinencephalon and the olfactory nerve were bilaterally absent but the chiasma opticum and pituitary gland were present and appeared to be normal (Fig. 2c). The anterior cerebral artery could not be identified but all other vascular structures had a normal appearance. Coronal brain sections demonstrated the absence of the inter-hemispheric fissure of the frontal lobes, frontal parts of the corpus callosum and the lateral ventricles (Fig. 2d). The more posterior parts of the corpus callosum and ventricles appeared normal as well as the thalamus, striatum, brainstem, cerebellum and occipital lobes.

## Discussion and conclusions

We report the first case of pancreatic agenesis occurring simultaneously with HPE. Although dysmorphic, the patient did not show any other defects compatible with known clinical syndromes associated with pancreatic agenesis and did not have mutations in any of the genes where mutations have been associated with pancreatic agenesis. Neonatal diabetes including pancreatic agenesis has been associated with abnormal brain development. In many of these patients a specific monogenic defect was identified in genes encoding transcription factors that are key during pancreas/beta cell development [7, 9, 10, 16, 23, 24]. Although HPE can have many causes other than genetic defects, the absence of the pancreas in the current case report strongly suggests a monogenic defect. This is underscored by its similarity to the reported cases of PTFA1 defects [7, 20], which was not the cause of the defects seen in the present patient. The severity and combination of the underlying abnormalities suggest an early defect during embryonic development, before budding of the pancreas, possibly at the end of gastrulation, when HPE develops and the ventral endoderm is in close vicinity of the developing forebrain. Disruption of a factor at this stage required for both endoderm specification and later pancreas development as well as development of the forebrain may explain the phenotype we observed.

Pancreas formation during embryonic life is under control of a complex network of transcription factors. This developmental program is providing landmarks for those who aim to recapitulate beta cell formation from human stem cells and provide unlimited supply to treat type 1 diabetic patients. Unique patients have proven to be an outstanding opportunity for the identification of novel transcription factors involved in beta cell development and the present case may help identify a novel key regulator in beta cell development.

## Abbreviation

HPE: Holoprosencephaly
